# Bin1 regulates synaptic endocytic trafficking compromised by Alzheimer’s genetic risk variants

**DOI:** 10.1002/alz.087074

**Published:** 2025-01-03

**Authors:** Claudia G. Almeida, Mariana Afonso Barata, Catarina Perdigão, Jose Ramalho

**Affiliations:** ^1^ CEDOC ‐ Nova Medical School ‐ Universidade NOVA de Lisboa, Lisboa Portugal; ^2^ Max Planck Institute for Multidisciplinary Sciences, Göttingen Germany

## Abstract

**Background:**

Alzheimer’s disease (AD), an untreatable synaptic disorder, is the most frequent cause of dementia. It is still unclear which mechanisms drive the early synapse dysfunction in the most common late‐onset AD (LOAD). The second most important LOAD risk gene identified, *BIN1*, is an endocytic regulator. We implicated Bin1 in BACE1 recycling with Bin1 loss of function, increasing axonal beta‐amyloid42. Since endocytic trafficking is essential for synapse function, we hypothesize that Bin1 may regulate synaptic endocytic trafficking, which may be disrupted by Bin1’s loss of function or beta‐amyloid42. We aim to dissect Bin1 causal mechanisms of synaptic dysfunction to assess how the risk for LOAD increases.

**Method:**

Synaptically mature mouse primary cortical neurons and brain hippocampus were used to investigate Bin1 synaptic endocytic localization with immunofluorescence and synaptosomes. Using knockdown, overexpression, and rescue approaches the impact on synaptic endosome size and synaptic density was determined using super‐resolution fluorescence microscopy and quantitative subcellular image analysis.

**Result:**

Bin1 was enriched in synaptic vesicle brain fractions, supporting its presynaptic function. Unexpectedly, Bin1 is enriched proximal synapses, identified as inhibitory GABAergic presynaptic terminals in primary neurons and the brain. Notably, Bin1 down‐regulation specifically reduced inhibitory synapse density and increased neuronal excitability. Bin1 loss increased inhibitory synaptic vesicle release and endocytosis, exhausting presynaptic endosomes. Interestingly, the impact on inhibitory synapses is independent of beta‐amyloid production. Moreover, Bin1 LOAD mutations interfere with Bin1 function and are likely to cause loss of function.

**Conclusion:**

Bin1 is an inhibitory presynaptic protein. The LOAD mutations may cause Bin1 loss of function, affecting inhibitory synapses. Losing inhibitory transmission may turn excitatory neurons hyperactive, accelerating the loss of excitatory synapses. In the future, we aim to dissect the underlying mechanisms by which Bin1 mutations interfere with Bin1’s role in synaptic vesicle mechanisms at inhibitory presynaptic terminals.

